# State-Run Dating Apps: Are They Morally Desirable?

**DOI:** 10.1007/s13347-024-00719-x

**Published:** 2024-02-22

**Authors:** Bouke de Vries

**Affiliations:** Department of Philosophy, Ghent University, Ghent, Belgium

**Keywords:** Dating apps, Love, Romantic relationships, Tinder, Grindr, Gamification, Addiction, Birth rates, States, Political philosophy

## Abstract

In a bid to boost fertility levels, Iran and Japan have recently launched their own dating apps, with more countries likely to follow. The aim of this article is to consider whether state-run dating apps are morally desirable, which is a question that has not received any scholarly attention. It finds that such apps have at least two benefits that collectively, if not individually, render their introduction to be welcomed provided certain conditions are met. These benefits are that they are better placed than commercial dating apps such as Tinder, Bumble, and Badoo to (i) help people find lasting love and to (ii) protect individuals from spending too much money and/or time on online dating. Several objections are discussed and shown to be unconvincing as arguments against state-run dating apps *tout court*, including the objection that for states to offer their own dating apps is unduly expensive; the objection that it gives them too much power; and the objection that they should invest in creating offline opportunities for meeting potential partners instead.

## Introduction

A lot of early-stage dating has moved to dating apps, which are smartphone programmes used for making initial contact with potential romantic partners and/or partners for casual sex that differ from traditional dating websites in that they track users’ geographical locations; feature user profiles that are heavily picture-based as opposed to text-based; and require individuals to evaluate these profiles by either swiping left to express a lack of interest in someone (dislike) or right to express interest (like), whereby users who mutually like each other’s profiles (a phenomenon known as ‘matching’) become able to communicate through the app’s chat function (Rosenfeld, 2018; Schwartz & Velotta, 2018). For example, almost half of young US adults aged 18-29 reports having used a dating app (Pew Research Center, 2020), whereas in the United Kingdom, it was found that even before the COVID-19 pandemic during which the use of online dating services increased (Wiederhold, 2021), more relationships among 18-35 year olds were initiated online (23%) than were initiated at work (20%); via a mutual friend (19%); or at a bar, pub, or club (17%) (Sasidhara et al., 2018). Given their already considerable and rising popularity – for example, it is estimated that by 2035, more British couples will have met on dating apps than in real life (Sasidhara et al., 2018) – it should come as no surprise that states with below-replacement fertility have started to take an interest in these apps and that Iran and Japan now offer their own dating apps in a bid to raise the national birth rate (Reuters, 2021a; The Economist, 2019).

In this article, my aim is to consider whether state-run dating apps are morally desirable, which is a question has not received any scholarly attention.^1^ I find that such apps have at least two benefits that together, if not individually, render their introduction to be welcomed provided certain conditions are met. (Whether these conditions are, or could foreseeably be, met within various contemporary societies requires in-depth empirical research that I cannot begin to undertake here; that said, I am reasonably optimistic that the answer is affirmative for those with strong institutional and legal safeguards against abuses of governmental power, such as the Scandinavian countries.) These benefits are that state-run dating apps are better placed than commercial dating apps such as Tinder, Bumble, and Badoo to (i) help people find lasting love and to (ii) protect individuals from spending too much money and/or time on online dating (sections 2and 3). Several objections are discussed and shown to be unconvincing as arguments against state-run dating apps *tout court*, including the objection that for states to offer their own dating apps is unduly expensive; the objection that offering such apps gives them too much power; and the objection that they should invest in creating offline opportunities for meeting potential partners instead (section 4). The final section concludes (section 5).

## Problems with Commercial Dating Apps

To start our investigation, we should begin by noting that it is far from evident that there are ever contexts where states should be offering their own dating apps as I believe there are. Given that the development and maintenance of such apps consumes resources that could be spent on things like policing, housing, and education or simply be left to tax-payers to spend as they see fit and that there currently exists a range of commercially-owned dating apps (e.g. Tinder, Bumble, Match.com, OK Cupid, Badoo, HappyPancakes), for states to make their own dating apps available may be deemed a waste of public resources. This section begins to question this notion by suggesting that there are at least two major problems plaguing commercial dating-apps. In the next sections, I will then go on to argue that state-run dating apps are less susceptible to these problems, before proposing that the distinct challenges raised by such apps do not always seem fatal.

### Gratuitous Prolongments of People’s Search for Love

One of the problems with commercial dating apps is that although their algorithms are kept secret (Klincewicz et al., 2022; Schwartz & Velotta, 2018, p. 127), there are strong grounds for suspecting that they are not optimized for helping users find lasting love, which many are seeking on their platforms despite apps such as Tinder having somewhat of a reputation for being solely about hook-ups (Timmermans & De Caluwé, 2017).To appreciate this, three observations are in order. The first is that that there are handsome profits to be made within the dating app-market, whose total revenue has grown continuously since 2015 and is estimated to have reached almost 5 billion USD in 2022 (Curry, 2023). The second observation is that being commercial entities, dating apps companies participate in this market to turn a profit whatever other objectives they might have, which many of them do. For example, Bumble is estimated to have a revenue of circa 3.4 million USD per 1 million users ahead of Tinder at circa 3.0 million and Grindr at circa 2.9 million (Morahan, 2023). The third observation is that this objective is undermined when users establish romantic relationships. This is because once such relationships are forged, many people delete their account and thereby reduce the revenue that is generated through one or more of the following sources:

- The sale of premium membership fees. The level of these fees tends to vary depending on factors such as the type of membership that is bought, the duration of the membership, and the median income of the country in which it is bought (Consumers International, 2022). In the US, premium membership fees for Tinder, which is the most widely used dating app in the world with an estimated 75 million monthly active users (World Population Review, 2023), can be anywhere between 7,99 USD (Tinder plus) and 29,99 USD (Tinder Platinum) for a one-month subscription (VidaSelect, 2023), but might rise to 500 USD in the future as the company announced in 2023 that it was developing a new subscription for the most affluent of users (Tinder Vault) (Neuts, 2023). Benefits of premium memberships might include, but are not necessarily limited to, a higher number of profiles that one can see and/or like; the ability to see which other users liked one’s profile without having to like their profiles first; the ability to contact other users without having to match first; the ability to backtrack in case one disliked a profile by mistake; the ability to use more fine-grained search criteria; the ability to use a limited number of visibility boosts that temporarily show one’s profile to a higher number of users; the ability to contact matches beyond a given time-limit;^2^ and the removal of adds (Beck, 2021; Klincewicz et al., 2022; Schwartz & Velotta, 2018; Vágó, 2022).
- The sale of add-ons that according to dating app companies can aid users in their quest for love and/or hook ups. Visibility boosts are one example of such add-ons, which in addition to being included in premium memberships by dating apps such as Tinder and Bumble are often sold separately. For instance, in the US, Tinder sells ‘Boosts’ that allow you to ‘be one of the top profiles in your area for 30 minutes’ and to ‘get up to 10x more profile views while boosting’ for 8 USD (*Boost*, n.d.) as well as ‘Super Boosts’ that make it possible for you to ‘cut to the front and be seen by up to 100x more potential matches’ during a 180 minute-period for 30 USD (*Super Boost*, n.d.) (Branson, 2023c). Another popular type of add-on are so-called ‘superlikes’, which enable users to signal that they are especially interested in someone’s profile (Tseng, 2022). They are sold by Tinder for roughly one USD per like with prices being somewhat lower for those who buy them in packages (Branson, 2023a, 2023b, 2023c), whereas Bumble also offers superlike subscriptions that range from a weekly subscription of 8 USD to a lifetime subscription of 139.90 USD (Branson, 2023a).
- The sale of user-data to third parties. A 2020 report found that several of the most popular dating app companies, including Tinder, OKCupid, and Grinder, have been selling personal data of Android-users to other companies, which included information about, *inter alia,* users’ sexual orientations, political views, drug-use histories, and real-time locations (Forbruker, 2020).^3^
- The sale of advertising space. While dating app companies such as Tinder and Bumble claim that advertising accounts for a comparatively small portion of their profits (Goldfine, 2021), other companies are known to rely more heavily on it. For example, Happy Pancake, one of the largest dating apps in Sweden, does not have paid subscriptions or add-ons and reportedly derives most of its revenue from adverts (Appscrip, 2022).^4^

What is pertinent for us is that the fact that these forms of revenue account for the large majority of profits within the dating app industry (Klincewicz et al., 2022) and all depend on people actively using dating apps makes it highly probable that, notwithstanding the secrecy surrounding their algorithms, many of these apps are designed to gratuitously prolong people’s search for lasting love (cf. First, 2018, pp. 548–549; Klincewicz et al., 2022, p. 560). By ‘gratuitously’ I here mean means that love-seeking users are kept on the app longer than they would be if, drawing on the vast amounts of personal data that is being collected by the owner companies, these companies presented them with profiles and profile orderings that were optimized for the goal of forging stable romantic relationships.

One direct piece evidence of such unnecessary prolongments is provided by reports of dating app companies using deception to maintain user engagement, for example by deploying fake profiles operated by chatbots or by their paying real individuals (usually women) to feign interest in specific users (usually men) (Gieselmann & Rasch, 2021; Hu et al., 2021; Porter, 2022). However, even when no deception is being used, there are ways in which such companies can, and most likely do, gratuitously extend many people’s quest for lasting love, which may include intentionally presenting them with profiles of individuals with whom they are unlikely to establish (long-term) romantic relationships and showing promising profiles to them at inconvenient times, such as when they have little time to go on dates or when they have just started to chat to or date with another, less suitable person.^5^

At this point, it should be asked: why care about people’s quest for lasting love being made longer than they would if their dating apps were optimized for finding lasting love? Whereas there are different reasons (more will be discussed in the next section), the one I want to concentrate on here is that gratuitously prolonging such quests for years or even months can, and often will, have considerable costs for them personally. To appreciate this, it should be observed that romantic relationships on average have a significant positive impact on people’s wellbeing, including that of women (Chen et al., 2023; Ge et al., 2020; Londero-Santos et al., 2021), with some studies finding that married people enjoy a 30 percentage point happiness advantage over their unmarried counterparts (Peltzman, 2023). Besides providing us with valuable goods such as emotional and physical intimacy (cf. Helm, 2021), such relationships constitute a major buffer against chronic loneliness (Dykstra & Fokkema, 2007; Flora & Segrin, 2000; Luhmann & Hawkley, 2016), which is important given that chronic loneliness contributes to depression (Cacioppo et al., 2010); dementia (Holwerda et al., 2012); and poor physical health (Aanes et al., 2010) and is a widespread phenomenon. For example, even prior to the COVID-19 pandemic, surveys from Europe and North-America reported that 20 to 35 percent of adults between the ages of 65 and 79 said that they are frequently lonely, a figure that goes up to 40-50 percent among those aged 80 and above (Dykstra, 2009). What follows is that although being in a long-term relationship is not everyone’s path to happiness and, even if it is, some individuals are worse off within their current relationship than they would be if they were single – think, for instance of those caught in abusive relationships (NCADV, 2020) – the considerable value that such relationships have for many *provide strong reasons for wanting people to have access to dating apps that are optimized for helping them to establish long-term relationships*^6^ even if these reasons are conditional on some of the ills associated with said relationships being addressed by e.g. ensuring that people have adequate opportunities to report domestic abuse and are entitled to equitable divorce arrangements.

#### Overspending Money and Time

The other problem with commercial dating apps I wish to zero in on – there might be more – is that their design poses a substantial risk that at least some users will spend too much money and/or time on their platforms. The risk of overspending money, understood as devoting a larger share of one’s income to online dating than one can afford taking into account one’s basic needs (e.g. housing, food, medicine), is best illustrated by the ability of users on most of these apps to purchase visibility boosts whose cost can take a hefty financial toll of users that quickly exceed those of a premium membership. For example, if an American Tinder user were to purchase only two superboosts a month at 30 USD each, they will already pay double the price of the most premium membership (platinum) which is also roughly 30 USD. Since such a boost only last 180 minutes, there is nothing stopping such a user from spending hundreds or even thousands, of Dollars on such boosts every single month.

Now, although I am unaware of any empirical research into the amounts of money spend on boosting by different groups of users, there is every reason to worry that some users are spending more on this type of add-on on than is good for them. For as I discuss in more detail elsewhere (self-citation omitted), the use of visibility boosts meets all the criteria for being a (non-conventional) form of gambling defined as ‘the act of wagering or betting money or something of value on an event with an uncertain outcome with the intent to win more money or things of value than was wagered’ (Winters et al., 2012, p. 18). Not only can buyers of such boosts win something that has value to them, namely additional likes that are marked on dating apps such as Tinder and Bumble with small icons to ensure that users know it was the visibility boost that landed them the like, how many additional likes they receive (if any) tends to vary from one boost to the next (*[Guys] How Many Matches Do You Get from a Boost?*, 2018), which is a phenomenon known as ‘variable reinforcement’ that plays a key role in causing addiction (M. Brooks, 2019). In fact, a closer look reveals that the use of visibility boosts is *structurally similar to the most addictive forms of gambling* such as slot machine games and the use of loot boxes, which are digital containers of randomized virtual items such as virtual football players and virtual weaponry whose sale has recently been found to have broken anti-gambling legislation in Belgium (Drummond et al., 2020, p. 986; Kansspel Comissie, 2018), by virtue of having low event horizons (i.e. people quickly find out whether their wager was successful) (Harris et al., 2021; Linnet et al., 2010).^7^ However, it is not just those with proclivities for developing gambling addictions who are at risk of overspending on visibility boosts and other add-ons such as superlikes (the costs of which can also add up, with Tinder charging roughly 1 USD per like and Bumble selling superlike subscription for 8 USD per week). The same is true of users who are lonely, as studies show that loneliness combined with a preference for online communication is conducive to compulsive dating app-use (Coduto et al., 2020), as well as of the sizable group of individuals who report using dating apps to receive validation from other users (Alexopoulos et al., 2020; Timmermans & De Caluwé, 2017).^8^

In addition to raising financial risks for certain types of users, then, commercial dating apps seem to be designed in ways that cause certain users to spend *too much time* on their platforms.^9^ By ‘too much time’, I here mean that someone is active on such apps for a greater number of hours than they want to upon reflection and/or than serves their mental health (and research by Her and Timmermans (2021) indicates that people’s wellbeing tends to be compromised by extensive dating app-use), which is a fate that seems to have befallen many. For example, both Tinder and Badoo report that, on average, their users spend circa 90 minutes a day on their apps divided over 10/11 log-ins (Badoo, 2016; Thomas et al., 2023), with some surveys suggesting that dating-app addiction is a widespread phenomenon (Eharmony, 2023). What is apposite for us is that rather than being an inevitable side-effect of dating apps, commercial dating apps *actively promote such excessive use* by gamifying their apps and incorporating non-conventional forms of gambling into their design such as the aforementioned use of visibility boosts (Abolfathi & Santamaria, 2020; de Vries, 2023; Klincewicz et al., 2022, pp. 556–559).^10^

## Comparative Benefits of State-run Dating Apps

Of course, just because there are problems associated with commercial dating apps, it does not follow that state-run dating apps are ever desirable. To establish this conclusion, one other thing that needs to be shown is that *such apps are more likely to stay clear of these problems than their commercial counterparts*, which will be the aim of the current section.

The reason why state-run dating apps appear less vulnerable to the inefficiency problem is that the interests of states and those of dating app users seeking stable romantic relationships are *more aligned* than the interests of dating companies and said love-seeking individuals respectively, which helps to explain why several countries have started to make their own dating apps available. Having witnessed there to be a significant misalignment between the latter already, what is pertinent for present purposes is that the interests of the former seem to largely if not fully converge. Without attempting to offer an exhaustive list of ways in which the formation of stable romantic relationships among members of a society may benefit states, one includes the fact that it can help them to save on health-care related expenses via the protection that such relationships offer against chronic loneliness, which can take a heavy toll on the public purse. In the UK, for instance, it is estimated that ill health caused by chronic loneliness costs employers £2.5 billion every year (HM Government, 2018), whereas in the US, Medicare is estimated to spend annually an additional $134 on a socially isolated older adult compared to an older adult who is not socially isolated (AARP Foundation, 2018). Other benefits include the fact that having such relationships increases people’s chances of marrying, which has been shown to reduce levels of criminal behavior and aggression among men (Burt et al., 2010; Sampson et al., 2006), as well as their chances of producing offspring, which can counteract the economic harms of below-replacement fertility that many countries across the world are experiencing (Lucero-Prisno III et al., 2022; United Nations, 2020).^11^ (Notice that these benefits for states exist *irrespective* of whether we conceive of the state as a corporate entity whose interests can be independent of those of any of its constituent members (List & Pettit, 2011) or as simply the sum of the interest of its officials or perhaps of those of the wider public as public interest theory maintains (Hantke-Domas, 2003). Given that a prosperous economy and social order enhance the state’s ability to maintain itself and exercise its power, it looks like the abovementioned consequences will be beneficial to states on both the corporate conception and the conception on which states interests are reducible to the interests of their officials. At the same time, these outcomes are ones that will normally serve the interests of those living within their societies, which would mean that state interests are also promoted on the third conception.)

In response, it might be argued that even if commercial dating apps seek to make profits first and foremost, by selling paid add-ons, they remain better positioned to help users find lasting love than do state-run dating apps that do not offer these functions. This is because, the argument goes, those seeking stable romantic relationships will be able to do things like shine a spotlight on their profiles by boosting; indicate that they are especially interested in particular users by superliking their profiles; and reverse any accidental left-swipes on users who might make for suitable partners, all of which will help them to achieve their goals.

There are two problems with this line of reasoning. One is that it is not evident that offering certain paid add-ons is necessarily in the interest of dating app-users seeking long-term relationships. Consider visibility boosts. Insofar as it is mostly individuals looking for hook-ups and short-term relationships who are purchasing these boosts, it would seem that, although any given user looking for lasting love might stand a better chance of finding a suitable partner by boosting than by refraining from this (I say ‘might stand a better chance’, as it is conceivable that their willingness to pay for such boosts will cause dating-app providers to try to keep them on their platforms longer in the hope that they will purchase more boosts, for example by showing their profiles to less suitable users; cf. de Vries, 2023), they would be better off still were such boosts completely unavailable as this would raise their per capita visibility. Likewise, if (almost) all users on a given dating app are looking for long-term relationships, using visibility boosts may not lead to an increase in the number of such relationships as they would simply be competing against each other.

However – and this brings us to the other, deeper, problem – even if their dating goals are served by being able to send superlikes, backtrack in case they accidentally disliked a profile, etc., the current argument is predicated on a false dilemma as states could give them such options *for free*. For example, rather than requiring users to pay for bundles of superlikes, they could simply give each user five superlikes per week and always allow backtracking.

Before moving on, I should highlight that *if* it turns out that some items such as superlikes are more likely to be bought by users looking for lasting love, such that offering these items for free is likely to undermine their interests, states could decide to offer said items (and only them) for a fee. Alternatively, or in addition, they could decide to exclusively allow users who have indicated that they are looking for long-term romance to send such likes, as well as bestow other privileges on them to increase the probability that committed relationships will materialize. For instance, they might choose to restrict access to visibility boosts to these individuals or permanently give them at least somewhat greater visibility on the dating app-platform compared to users with other motivations. What apposite for present purposes is that *such measures too* are less likely to be taken by commercial dating app-providers given their comparatively strong financial interests in user retention, lending further credence to the notion that state-run dating apps could be desirable under certain circumstances.

What about the ability of state-run dating apps to avoid the overspending time and money-problem? Here too, I believe that such apps fare better than their commercial counterparts. The reason is that as non-profit organisations, governments lack interests in their inhabitants becoming addicted to dating apps or in them spending huge sums of money on their platforms that they can ill afford. As such, they are less likely to design apps that are (highly) addictive and that include paid add-ons such as visibility boosts and superlikes. Furthermore, even if they decide to include (some of) them, they remain more likely to cap the amount of money that users can spend on said add-ons and to refrain from aggressively marketing these to users as many commercial dating apps do. For example, Tinder sends messages during peak hours telling users that ‘now would be a good time to boost’, whereas Bumble regularly sends ones proposing that users superlike specific profiles.^12^

Some might say that the mere availability of state-run dating apps without paid add-ons does not stop people from overspending on commercial dating apps. To prevent this, states would need to follow in Iran’s footsteps by not simply launching their own dating apps but also banning all private competitors (Reuters, 2021b). But that, it might be plausibly argued, would involve an unacceptable level of state interference with the market.

My rejoinder is that although state-run dating apps are not a panacea against overspending on dating app services as long as commercial dating apps remain available, it does not follow from this that such apps are not needed as a safeguard against this risk. On the contrary, by offering those who do not wish to be tempted to buy visibility boosts, superlikes, etc. a dating app that either does not sell these items to them or one that limits the amount of money that users can spend on them and that refrains from marketing them aggressively, their existence still appears to be an improvement upon the status quo. To bring this out, one might draw an analogy with the presence of supermarkets within a society where either no alcohol is sold or where there are restrictions on the types of alcohol that can be sold (as found in Sweden where supermarkets are forbidden from selling beverages with more than 3.5 percent alcohol) and/or on the places where alcohol can be stored (as found in New Zealand, where its Sale and Supply of Alcohol Act of 2012 prohibits supermarkets from placing alcohol within prominent areas such as check-out counters). Even if there remain opportunities for people within such societies to purchase (hard) alcohol, the availability of these types of supermarkets still seems desirable, as it reduces the amount of self-restraint alcohol addicts must exhibit to avoid indulging their addiction while going about an activity that is important for fulfilling a basic need (the need for adequate nutrition). Similarly, what I am proposing is that the availability of state-run dating apps that either lack paid add-ons or that cap the amount of money that users can spend on said add-ons and regulate how they may be promoted is desirable *even if* there remain commercial dating apps that do not satisfy either disjunct. This is because the availability of such apps makes it significantly easier for people to utilize mobile dating – which in the same way supermarkets have become one of the main way gateways to nutrition has become one of the main gateways to a romantic relationship – *without* being tempted to overspend on various add-ons, which we saw may be challenging for users with proclivities for addictive gambling as well as for those suffering from intense loneliness and maladaptive needs for external validation.

## Objections to State-Run Dating Apps and Some Rejoinders

Taking stock, I began this article by suggesting that there are at least two major problems plaguing commercial dating apps, namely that their design seems suboptimal for those seeking lasting love and comes with significant risks of users spending excessive time and/or money on their platforms, before arguing that state-run dating apps are less likely to suffer from these flaws. Yet, to demonstrate that these latter apps are at least sometimes desirable, we must also show there to be no fatal objections to them, which will be the task of the present section. To do so, three objections will be addressed that I consider to be the most promising.

The first of these maintains that it is *too expensive for states to develop and maintain their own dating apps*, which it was noted consumes resources that could be funnelled to other social goals (e.g. housing, education) or simply be left to taxpayers to spend as they deem fit. The problem with this objection is that even when we set aside the non-financial benefits of introducing the kinds of state-run dating apps defended within the previous section, the costs of these apps are likely to be offset by their expected economic benefits. According to various app-developing companies, the costs of building a dating app range between 25,000 USD for the most basic app to 1,25,000 for an app with all-inclusive features (Chavda, 2023; Crowdbotics, 2023; Mickiewicz, 2020; Saxena, 2022; Shakuro, n.d.), with most estimates being between 50,000 USD and 150,000 USD. As far as maintenance costs – e.g. hosting charges, API integration, bug fixing, IT support – are concerned, software engineer and writer Attila Vágó estimates the total amount to be under 1 USD per user per month, while Maharsi Pancholi, an employee at app-development company Brainvire, puts the figure at 5 percent of the developing costs per annum (Pancholi, 2022). Turning to the potential savings of having dating-apps that are optimized for finding lasting love, I have mentioned already that chronic loneliness, which romantic relationships are one of the best bulwarks against (Dykstra & Fokkema, 2007; Flora & Segrin, 2000; Luhmann & Hawkley, 2016), is estimated to costs UK employers approximately £2.5 billion annually (HM Government, 2018) and that Medicare is estimated to annually spend approximately 134 USD more for each socially isolated older adult compared to an older adult who is not socially isolated (AARP Foundation, 2018). When we add to this the high economic costs of male anti-social behavior which marriage helps to mitigate (López-Sánchez et al., 2019), including that of gender-based violence which according to the European Institute for Gender Equality costs approximately €366 billion a year across the EU (EIGE, 2023), and those of sub-replacement fertility (Lucero-Prisno III et al.,^ 2022^), it looks like state-run dating apps will already be cost-effective if they produce very small, and it appears feasible, increases in the number of stable romantic relationships, say of 1 or 2 percent.

Some may concede this but argue that it is *simply too dangerous* for states to have their own dating apps. One risk here is that they will misuse the large amounts of personal information that many users share on these apps. For example, it might be argued that Iran’s population would be better off if there were only commercially owned dating apps available, which we saw are currently outlawed within this country, given that its oppressive regime might utilize user-data gathered on the state app to identify and crack down on political dissidents and apostates (apostacy from the Shia faith being a capital offense). Another risk of state-run dating apps is that governments will deploy these apps for objectionable forms of social engineering.^13^ For example, to create or consolidate an apartheid-like system, they could (secretly) design their algorithms in ways that reduce the chances that members of particular ethnic groups will mix. Alternatively, or in addition, they might devise algorithms that lower the probability that political dissidents or disliked sexual and ethnic minorities will reproduce or simply have large families, which would be less radical attempts to prevent certain groups from reproducing than the forced sterilizations that were committed in countries such as Germany, Sweden and the US during parts of the 19^th^ and 20^th^ centuries (e.g.Bashford & Levine, 2010; Reilly, 2015) but still deeply troubling interventions.

My reply is that while these dangers are real, they only establish that states that are likely to put dating apps to *nefarious uses* should avoid becoming or remaining providers of this type of technology. While this might apply to Iran, it is not obvious that it does to states that are reasonably just in that they broadly respect their citizens’ individual freedom and equality, as I take it many contemporary liberal democracies do (cf. Rawls, 2001). Furthermore, insofar as significant risks exist even within these societies, there are measures that states can, and I assume should, take to reduce these risks and/or to mitigate the harms of their occurrence so that the relevant dangers become tolerable when set against the large personal and collective benefits that we saw state-run dating apps can have. Such measures include, but are not necessarily limited to:

- Introducing privacy protection laws that limit the state’s ability to access and store user-data, such as the EU’s General Data Protection Regulation of 2018.
- Enacting transparency laws that require the algorithms of state-run dating apps to be published and to be explained to laypeople (Kim & Routledge, 2022).
- Appointing agents (e.g. ombudsmen, external monitoring bodies) tasked with identifying misuses of state-run dating apps (Reif, 2004).
- Extending legal protections to whistle-blowers (Ceva & Bocchiola, 2019).
- Allowing commercial dating apps to co-exist along state-owned ones^14^ to mitigate the harmful effects of misuses of the latter.^15^

A final objection says that rather than making their own dating apps available, state should be *helping their citizens to find romantic partners offline*, for example by subsidizing in-person speed-dating events or by helping to keep afloat venues where most young people would meet until recently, such as discotheques and bars (Marston et al., 2020; Rosenfeld, 2018, p. 201). Some of those who hold this view may do so because they believe that online dating hardly ever results in long-term romantic relationships. Yet, although research on this topic is still in its early stages given the recent pedigree of dating apps, there exist already several studies and surveys suggesting that this belief is mistaken. We saw already that wanting a long-term romantic relationship is among the most common motives of dating-app users even on apps associated with hook-ups, such as Tinder (Timmermans & De Caluwé, 2017), and that that even before to the Covid-19 pandemic, more relationships were initiated online among 18-35 year Brits than anywhere else, with some forecasts suggesting that more British couples will have met on dating apps by 2035 than in real life (Sasidhara et al., 2018). Further evidence against such scepticism about the efficacy of mobile dating is provided by the following studies:

- Selterman and Gideon (2022) found that experiences of romantic attraction in dates that were initiated offline were the same as those initiated on dating apps among a US sample.
- Barrada et al. (2021) found no differences in long-term relationship orientation between those who use dating apps as opposed to those who do not among a Spanish sample.
- Research by Timmermans and Courtois (2018) reports that more than a quarter of Tinder-initiated dates among a Belgian sample led to a committed relationship (Timmermans & Courtois, 2018).
- Potarco (2020) found that non-residential Swiss couples who met through mobile dating have stronger interests in cohabitation than their counterparts who met offline.
- This last study also shows that Swiss couples who met via dating apps more generally do not experience lower relationship satisfaction or life quality than those who met offline. (While Sharabi and Dorrance-Hall (2024) do report lower marriage quality and stability for those who met on dating apps in the US, their effect sizes are modest and the reported marital quality is still high among both groups.)

Another possible reason for wanting states to stay out of the mobile dating market and instead focus their efforts on helping love-seekers to meet offline is that this circumvents several problems associated with the use of dating apps. One might think, for instance, of the fact that people feel less inhibited to engage in sexist, racist, and other obnoxious speech online than in real life (Carlson, 2020; Klincewicz et al., 2022, pp. 564–568); the fact that identity fraud is easier to commit on such apps than it is in person given the opportunities for users to hide behind other people’s pictures, which is sometimes referred to as ‘catfishing’ (Simmons & Lee, 2020); and the fact that many queer places of sociability, such as gay bars and LGBTQ+ community centres, had to close over the past decade due to the popularity of Grindr in particular, which some blame for having raised levels of loneliness among sexual minorities (Powers, 2019).^16^

Two replies are in order. One is that although these are genuine problems, the fact that the demand for dating apps is unlikely to drop significantly anytime soon – on the contrary, some experts expect it to continue to grow for the foreseeable future (Sasidhara et al., 2018; Statista, 2023) – renders it doubtful as to whether investing in offline opportunities for love-seekers to meet could spark a widespread return to the old ways. The other thing to say is that it is unclear whether such a return would even be *desirable*, given that mobile dating has benefits vis-à-vis meeting offline that might well outweigh its drawbacks. Arguably the most important one is that it makes it considerably easier to find romantic partners for many people, who include night shift-workers; those working long hours (Schwartz & Velotta, 2018); those living in areas where there are few persons with their sexual orientation (Castro & Barrada, 2020);^17^ those with disabilities that impede their ability to leave the home (Marston et al., 2020); and those who are highly afraid to approach potential partners in real-life, perhaps because they are introverts or because they are very sensitive to rejection^18^ (Aretz et al., 2010; Orosz et al., 2016).

## Concluding Remarks

That concludes my qualified defence of state-run dating apps. As I have sought to show, state-providers of dating apps seem generally better placed to help individuals find long-term romantic partners and protect those at risk of spending too much money and/or time on online dating than commercial providers such as Tinder, Bumble, and Badoo. This is not simply because they lack the profit-seeking objectives that incentivize the latter organisations from designing their apps in ways that gratuitously prolong people’s quests for lasting love and cause some users to overspend money and/or time, although this is important. Regardless of precisely how state interests are construed, I argued that there is considerable overlap between the interests of love-seeking dating app-users and those of states due to the social and economic benefits associated with people having stable romantic relationships. I ended this article by defending state-run dating apps against (what I believe are) the most plausible objections to them, which asserted that for states to offer their own dating apps is unduly expensive; gives governments too much power; and constitutes a less desirable way of promoting lasting romantic relationships than investing in offline opportunities for meeting potential partners.^19^

To prevent misunderstandings about these findings, let me reiterate that I have not argued that dating apps should be offered by authoritarian states. Given the high risks that these forms of technology will be used by such regimes to e.g. persecute sexual minorities or engage in morally objectionable forms of social engineering, for example by making it more difficult for some ethnic or religious groups to find partners, people living in unfree societies might well be better off if their states do not participate in the dating app-market. This is true especially, but not exclusively, when all private competitors are banned as in Iran.

Another thing to highlight is that even if, as it appears, these risks are significantly lower in contemporary liberal democracies, particularly in those with robust legal and institutional protections against abuses of state power such as the Scandinavian countries, the troubling legacies of governmental interference with people’s private lives within many of these societies^20^ suggests that precautionary measures should still be taken to reduce them. For example, I proposed that whatever else may be required, it will be necessary to have transparency laws requiring the algorithms of state-run dating apps to be published and to be explained to the general public, as well as to allow commercial dating apps to exist alongside state-owned ones. While such measures cannot wholly eliminate the danger of misuse and its concomitant harms, it is important to observe that based on this article’s findings, *this cannot reasonably be expected*. For if I am right that the alternatives, namely having exclusively commercial dating apps within a society or having no dating apps at all, each carry high opportunity-costs – whereas the former gratuitously prolongs people’s quest for lasting love and causes some users to overspend money and/or time on mobile dating, which are outcomes that not only have significant personal costs but also societal ones, the latter was found to make it a lot more difficult for certain groups to find romantic partners, such as night shift workers and sexual minorities – then it seems to follow that, up to a point, this sort of risk will be a price worth paying.

My hope is that future research will investigate in more depth exactly where this point is located and how likely it is that various contemporary states will stay clear of it if they do not do so already.

## Data Availability

Not relevant (this paper has no associated date or materials)

## Footnotes

1 More generally, the topic of online dating is one that has been largely neglected by philosophers. To date, there are only four philosophical publications on this topic. One of these concerns an article by Nader (2020), who argues that there is compelling reason to think that even when commercial dating apps do not allow users to select people on the basis of their ethnicity or race as e.g. Grindr did until a few years ago (BBC, 2020), they are still likely to be presented with ethnically and racially biases recommendations – one that favor white people in particular – based on the collaborative filtering algorithms that many of these apps employ. Another concerns an article by Lexie Unhjem et al. (2021), who explore ethical issues pertaining to the use of dating apps by mental health professionals, such as in-app encounters with clients. The remaining publications consist of a book chapter by (Klincewicz et al., 2022), which details a range of morally salient impacts of dating apps, and an article by myself (self-citation omitted), which defends legal restrictions on the sale of visibility boosts on dating apps.

2 For example, Bumble requires users to send their first message within 24 hours of matching.

3 In some countries, this practice was found to have broken domestic privacy laws. For example, in 2022, Grindr was fined 7.7 million USD by Norwegian regulator Datatilsynet for selling sensitive use data (Arntz, 2021).

4 Besides these relatively direct forms of revenue loss, for people to cease using dating apps might affect the profitability of their owner companies in a more indirect way. This occurs when membership attrition renders their apps less attractive for both existing users and potential future ones due to there being fewer individuals to match with, which might lead to a further reduction in membership rates and thus initiate – from the perspective of these companies – a vicious circle.

5 Lest I be misunderstood, I am not denying that dating app-companies also have *pro tanto* interests in users finding lasting love. If no one ever did, their apps could come to be regarded as ineffective or simply as inefficient, which might undermine people’s willingness to sign up to them and be active on their platforms (thereby reducing revenue generated through advertising and the sale of user-data to third party companies) along with the willingness of users to pay for premium memberships and various add-ons. Rather than arguing that dating app companies are *always* seeking to maximize membership retention at the expense of people’ chances of finding lasting love, my claim here is more modest, namely that there are compelling reasons to expect that in a large number of cases, the search for such love is deliberately being extended even if there are limits to how far these companies try to extend it.

6 This is true especially when we consider that such relationships also have important societal benefits about which I will say more in the next section.

7 Notice that the fact that, unlike slot machine games, visibility boosts lack monetary prices does not preclude people from developing addictions to them. Perhaps the best way of illustrating this is to observe that loot boxes do not generally have monetary prizes or prizes that can be converted to money either yet have been shown to be highly addictive to a subset of buyers nonetheless (e.g. Brady & Prentice, 2021; G. A. Brooks & Clark, 2019; Drummond & Sauer, 2018; Zendle & Cairns, 2019).

8 For example, one young woman describes how her ‘sociopathic curiosity and appetite for constant validation is fueled by Tinder’s addictive function,’ which as she explains has led her to develop a habit of ‘consuming hundreds of profiles on boring journeys or in queues for a slow barista’ (quoted in Kent, 2015).

9 Of course, this distinction might not be entirely neat if we accept the dictum that ‘time is money’.

10 As one victim of what is sometimes called ‘doomswiping’ describes the phenomenon, ‘I open up the app with some kind of intention… but the swiping just becomes another way to stare at a screen and not think about anything’ (Lovine, 2020).

11 Another potential benefit of increases in the number of stable romantic relationships is that they may help to reduce housing shortages, as couples are more likely to live together than singles.

12 Another way in which purchases of add-ons can be promoted is by including a small number of them in premium memberships as Tinder and Bumble do with boosts and superlikes, which might create a desire among users to purchase these virtual items separately once they have used them all up. One might liken this strategy to the one used by drug dealers to get people hooked on their supply by giving away free samples to first-time users. One reason for thinking this could be an effective strategy is that the experience of using free versions of dating apps is often made quite miserable by e.g. exposing freemium users to lots of adverts; making it impossible for them to backtrack if they mistakenly disliked a profile; and hiding the profiles of users who liked them while informing them that *some* users swiped right on them (cf. Vágó, 2022).

13 I discuss this risk in more detail elsewhere; see (self-citation omitted).

14 Which, it was noted, Iran does not do.

15 It is true, as one of the reviewers of this journal has pointed out to me, that certain comparable measures might be taken to address the problems with commercial dating apps as examined in section 2. For example, such apps could be legally required to optimize their algorithms for the purpose of establishing long-term relationships as well as to limit the amount of money and/or time that users are able to spend on their platforms. While some of these measures strike me as defensible (e.g. imposing limits on the amount of money that users can spent on such apps and possibly also the amount of time they can spend on them, although this is more controversial), there seem to remain sufficient grounds for having state-run dating apps in societies where the risks of such apps being utilized for objectionable purposes are sufficiently low. As I discuss in more detail in other work (self-citation omitted), not only is the introduction of state-run apps less intrusive than requiring the algorithms of commercial dating apps to fit certain state-directed goals, such requirements are costly to police and might give governments an excessive amount of power over people’s private lives by putting the entire domestic dating app-market under their control. What is more, even if these objections are not fatal, the strong intellectual property rights-protections in Western countries render it unlikely that governments in this part of the world will be able to exert such control anytime soon (Laat, 2022).

16 As one gay man comments on this development, ‘it’s so much easier to meet someone for a hookup on Grindr than it is to go to a bar by yourself. Especially if you’ve just moved to a new city, it’s so easy to let the dating apps become your social life. It’s harder to look for social situations where you might have to make more of an effort’ (quoted in Hobbes, 2017).

17 To facilitate the search for individuals with minority gender identities and sexual orientations, OKCupid now offers 22 gender identity options to choose from, including ‘bigender’, ‘pangender, and ‘non-binary’ and 12 sexual orientations, including, ‘pansexual’, heteroflexible’, and ‘queer’, although that of a ‘sapiosexual’ was recently removed (*Gender and Orientation on OkCupid*, n.d.).

18 Which is much less explicit on dating apps as people are only notified when someone matches with them, not when they are being swiped left on.

19 Which, it bears stressing, does not entail that such investments should not be made. All it means is that they should not function as a *substitute* for state-run dating apps.

20 Which we saw in the most extreme cases involved forced sterilizations of individuals deemed unwanted.

## Declarations

### Authors’ Information

I am an associate professor in philosophy at Ghent University, interested in social relationships among other topics.
